# Association between serum calcium and 28-day mortality in critically ill patients with COVID-19: A retrospective cohort study from MIMIC-IV database

**DOI:** 10.1097/MD.0000000000046644

**Published:** 2025-12-19

**Authors:** Jianmin Qu, Yingxiu Huang, Lihua Shen

**Affiliations:** a Department of Intensive Care Unit, Tongxiang First People’s Hospital, Tongxiang, Zhejiang Province, China; b Department of Infectious Disease, Beijing Luhe Hospital, Capital Medical University, Beijing, China; c Department of Hospital Infection and Public Health, Tongxiang First People’s Hospital, Tongxiang, Zhejiang Province, China.

**Keywords:** calcium, COVID-19, MIMIC-IV, mortality

## Abstract

Although serum calcium levels are associated with the prognosis of various critical illnesses, their specific relationship with 28-day mortality in critically ill patients with coronavirus disease 2019 (COVID-19) remains unclear. This relationship has potential implications for patient management and prognosis assessment. This investigation examines the association between serum calcium concentrations and 28-day mortality among severely ill patients with COVID-19. This retrospective cohort analysis encompassed 806 intensive care unit admitted COVID-19 patients, utilizing data extracted from the Medical Information Mart for Intensive Care – IV (MIMIC-IV) 3.0 database. Information regarding vital signs, laboratory test results, and preexisting conditions was compiled to explore the association of serum calcium concentrations with 28-day mortality. This study comprised 806 critically ill COVID-19 patients, averaging 64 years of age, of which 59% were male. The 28-day mortality rate for this cohort was 27.7%. Preliminary analyses indicated a strong relationship between elevated serum calcium levels at admission and a lower 28-day mortality rate (hazard ratio [HR] = 0.71; 95% confidence interval [CI] = 0.61–0.82; *P* < .001). Following adjustment for various confounding factors, the correlation between serum calcium concentrations and decreased 28-day mortality retained statistical association (HR = 0.83, 95% CI = 0.71–0.97, *P* = .021). Stratification of calcium concentrations into tertiles showed that patients in the top tertile (T3) exhibited a markedly decreased risk of 28-day mortality versus individuals within the bottom tertile (T1) (model III: HR = 0.70, 95% CI = 0.50–0.99, *P* = .043). Subgroup assessments showed comparable results across distinct demographic groups. Elevated serum calcium concentrations at the time of admission were linked to a reduced 28-day mortality in critically ill patients with COVID-19, offering a new perspective on the role of calcium in the management and prognosis of COVID-19.

## 1. Introduction

Pandemics exert a significant impact on global economies and public health systems. The coronavirus disease 2019 (COVID-19) pandemic, which emerged in 2019, is among the most devastating pandemics in recent memory.^[1]^ On March 11, 2020, the World Health Organization declared the outbreak a global pandemic, presenting an unprecedented challenge that has had profound and widespread effects on the global economy and societal structures. By October 13, 2024, the severe acute respiratory syndrome coronavirus 2 (SARS-CoV-2) had led to over 776.6 million infections and 7.1 million deaths on a global scale.^[2]^ SARS-CoV-2 infection in individuals presents a spectrum of clinical outcomes, varying from asymptomatic carriage to critical respiratory distress necessitating high dependency or intensive care, often culminating in fatal outcomes.^[1]^ Critical respiratory distress is the predominant reason for intensive care unit (ICU) admission among patients in the critical phase of COVID-19. In addition, individuals in this phase often present with metabolic disorders, which may influence their clinical outcomes.^[3–5]^ Out of these metabolic abnormalities, it appears that metabolic acidosis is associated with a poorer prognosis.^[4,6]^

Calcium, an indispensable cation, plays a pivotal role in cellular functions, metabolic pathways, and signaling cascades, thereby substantially contributing to viral persistence and pathogenicity.^[7]^ Recent research has highlighted the assessment of serum calcium levels as a critical factor in understanding and managing various infectious diseases, sepsis,^[8,9]^ tuberculosis,^[10]^ acute osteomyelitis,^[11]^ and COVID-19.^[12]^ Previous research has indicated a notable occurrence of hypocalcemia in patients diagnosed with COVID-19.^[13]^ However, the connection between serum calcium concentrations and fatality risk among ICU patients with severe COVID-19 is not yet fully understood.

This cohort study evaluates the correlation between serum calcium concentrations and 28-day mortality in patients with critical conditions due to COVID-19 receiving intensive care. We hypothesize that there is an association between serum calcium concentrations and the 28-day outcome in terms of fatality in patients requiring intensive care for COVID-19.

## 2. Methods

### 2.1. Database

The data for this research were sourced from the Medical Information Mart for Intensive Care – IV (MIMIC-IV), version 3.0, a major ICU data repository, which is available at https://mimic.mit.edu/.^[14]^ The dataset encompasses data on 94,458 hospitalizations of critically ill patients treated at Beth Israel Deaconess Medical Center (BIDMC), Boston, covering the years 2008 to 2022.^[15]^ The dataset contains extensive data points, such as survival status, vital signs, laboratory tests, diagnostic details, and treatment protocols. The MIMIC database contains comprehensive, high-caliber data, increasingly utilized by researchers for academic studies.^[16,17]^ After successfully completing the online training and exam (Certificate ID: 56513391), Yingxiu Huang, one of the authors, was granted access to the database. Given the database’s safeguarding of patient information, formal consent was not necessary. This cohort study conformed to the STROBE guidelines (Strengthening the Reporting of Observational Studies in Epidemiology), thereby improving transparency in the reporting of observational studies.^[18]^

We performed a retrospective cohort analysis using the MIMIC-IV database (version 3.0), focusing on ICU admissions for the first COVID-19 episode. The collected data covered patient demographics, preexisting medical conditions, illness severity scores, and laboratory test results. The Charlson Comorbidity Index (CCI) and Sequential Organ Failure Assessment (SOFA), the tool for assessing illness severity, SOFA score of ≥ 5 as the criterion for severe stratification in our study.^[19,20]^ The comorbidities included sepsis, diabetes, hypertension, liver disease, chronic obstructive pulmonary disease (COPD), renal disease, asthma, and acquired immune deficiency syndrome (AIDS). The treatment measures included antivirus drug use with 5 days of ICU admission, corticosteroids (dexamethasone, hydrocortisone, prednisone, and methylprednisolone), heparin and its derivatives (heparin sodium, enoxaparin sodium, and fondaparinux sodium), vasopressor use, invasive mechanical ventilation (IMV) first day of ICU stay. The primary endpoint of this cohort study was mortality at 28 days.

### 2.2. Study cohort

#### 2.2.1. Criteria

Initially, we enrolled individuals diagnosed with COVID-19, with sequence numbers ranging from 1 to 5, hospitalized between 2008 and 2022. Diagnosis was confirmed based on the ICD-10 code “U071”. Subsequently, we limited our selection to individuals first hospitalized in the ICU. Ultimately, we confined our analysis to those whose serum calcium levels were recorded within the initial 24 hours post-ICU admission.

#### 2.2.2. Covariates

Data extraction via the MIMIC-IV database was performed using Structured Query Language (SQL), with the data subsequently stored in a PostgreSQL database. The dataset comprised patient demographics including age, gender, race, body mass index (BMI), vital signs. Biochemical and hematological data collected within the first 24 hours of ICU admission included initial measurements of metabolic and hematologic markers, such as white blood cell count, hemoglobin, platelets, red blood cell distribution width (RDW), lactate, and other relevant biomarkers. Patients presented with various comorbidities, including diabetes, hypertension, sepsis, liver disease, COPD, renal disease, asthma, and AIDS. Illness severity was quantified utilizing the CCI. Treatment measures included antivirus drug use with 5 days of ICU admission, corticosteroids, heparin and its derivatives, vasopressor use, IMV on the first day of ICU stay.

### 2.3. Statistical analysis

Individuals were stratified into 3 groups according to the tertile distribution of serum calcium concentrations. Descriptive statistics were used to summarize the characteristics. Continuous variables were summarized as mean ± standard deviation for normal distributions or as median with interquartile range for skewed data, whereas categorical variables were reported as percentages. Baseline characteristics were assessed by comparing continuous variables with the Student *t* test or Mann–Whitney test, and categorical variables with the chi-square test. Variables with under 40% missing data were imputed by the K-nearest neighbors approach.^[21]^

To assess the independent association of serum calcium levels with 28-day mortality, multivariable Cox proportional hazards regression models were used to estimate hazard ratios (HRs) and 95% confidence interval (CIs) for mortality risk. An extended Cox model was used to adjust for multiple variables, with the reference group defined by the lowest serum calcium concentrations. Model I: no adjustment; model II: adjustment for gender, race, age, and BMI; and model III: model II plus RDW, partial pressure of oxygen in arterial blood, hypertension, diabetes, sepsis, liver disease, COPD, renal disease, asthma, AIDS, CCI, antiviral drugs, corticosteroids, heparin and its derivatives, vasopressor, IMV. Trend tests were performed by using the median of each tertile as a continuous predictor. Furthermore, a comprehensive Kaplan–Meier analysis was performed to generate Kaplan–Meier curves, which were compared using the log-rank test. A meticulous analysis of subgroups was undertaken to explore whether serum calcium levels affect mortality across various subgroups.

Analyses were performed using R (version 4.2.2 http://www.R-project.org, R Foundation) and Free Statistics software (version 2.2.0).^[22]^ Statistical significance was assessed with a 2-tailed test and *P*-value < .05.

## 3. Results

### 3.1. Participants’ characteristics

**Figure 1.**
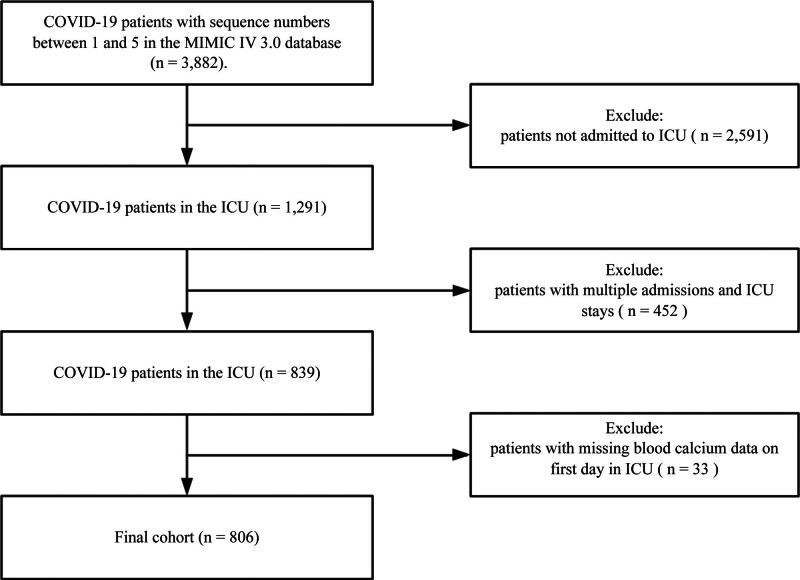
Flowchart of the study. COVID-19 = coronavirus disease 2019, ICU = intensive care unit, MIMIC-IV = Medical Information Mart for Intensive Care – IV.

After applying the predefined inclusion and exclusion criteria, 806 critically ill COVID-19 patients were included from the MIMIC-IV database (Fig. 1). The final analysis included 806 patients, 59% male, with a mean age of 64 years. Among these patients, 223 succumbed to the disease, yielding a 28-day mortality rate of 27.7%. A detailed summary of the demographic and clinical characteristics of all participants is provided in Table 1.

**Table 1:** Baseline characteristics of participants.

Variables	Total (N = 806)	Serum calcium (mg/dL)			*P*-value
		T1 (1.5–7.9) (n = 262)	T2 (8.0–8.4) (n = 243)	T3 (8.5–11.3) (n = 301)	
General characteristics					
Age (yr)	64.1 ± 16.5	62.4 ± 16.8	63.5 ± 16.2	66.0 ± 16.3	.031
Gender (male), n (%)	477 (59.2)	164 (62.6)	134 (55.1)	179 (59.5)	.233
Race					.221
White, n (%)	336 (41.7)	98 (37.4)	108 (44.4)	130 (43.2)	
Non-White, n (%)	470 (58.3)	164 (62.6)	135 (55.6)	171 (56.8)	
BMI (kg/m^2^)	31.1 ± 6.5	31.0 ± 7.7	31.1 ± 6.2	31.3 ± 5.5	.903
Vital sign					
Temperature (°C)	37.5 ± .9	37.7 ± 1.1	37.6 ± .8	37.4 ± .7	<.001
Heart rate (bpm)	104.0 ± 21.2	108.6 ± 23.9	103.0 ± 19.2	10.8 ± 19.6	<.001
Respiratory rate (bpm)	31.9 ± 7.1	31.8 ± 7.4	32.3 ± 6.8	31.8 ± 7.0	.650
Mean arterial pressure (mm Hg)	62.7 ± 14.4	58.9 ± 15.0	62.9 ± 13.4	65.8 ± 13.9	<.001
Pulse oxygen saturation (%)	89.0 ± 6.7	88.7 ± 6.7	89.3 ± 7.3	89.1 ± 6.0	.616
Laboratory parameters					
White blood cell (×10^9^/L)	1.0 (7.1, 14.3)	11.2 (7.6, 16.7)	9.4 (6.6, 13.6)	9.7 (7.2, 13.2)	.001
Hemoglobin (g/dL)	11.1 ± 2.5	1.1 ± 2.4	11.4 ± 2.3	11.8 ± 2.4	<.001
Platelet (×10^9^/L)	21.0 (152.0, 282.0)	187.5 (127.2, 258.5)	21.0 (152.0, 277.0)	224.0 (17.0, 30.0)	<.001
RDW	14.8 ± 2.3	15.1 ± 2.2	14.7 ± 2.3	14.6 ± 2.3	.018
PaO_2_ (mm Hg)	84.1 ± 28.0	84.6 ± 28.2	85.7 ± 32.1	82.3 ± 24.1	.334
Lactate (mmol/L)	1.7 (1.2, 2.7)	1.8 (1.3, 2.9)	1.6 (1.1, 2.3)	1.7 (1.2, 2.3)	.007
Commodities, n (%)					
Hypertension	492 (61.0)	141 (53.8)	150 (61.7)	201 (66.8)	.007
Diabetes	268 (33.3)	77 (29.4)	83 (34.2)	108 (35.9)	.248
Sepsis	491 (6.9)	195 (74.4)	156 (64.2)	140 (46.5)	<.001
Liver disease	18 (2.2)	5 (1.9)	6 (2.5)	7 (2.3)	.905
COPD	83 (1.3)	28 (1.7)	25 (1.3)	30 (10)	.961
Renal disease	165 (2.5)	48 (18.3)	44 (18.1)	73 (24.3)	.121
Asthma	67 (8.3)	19 (7.3)	25 (1.3)	23 (7.6)	.405
AIDS	4 (0.5)	0 (0)	2 (.8)	2 (0.7)	.461
Disease severity scores					
CCI	4.0 (2.0, 6.0)	4.0 (2.0, 6.0)	4.0 (2.0, 6.0)	4.0 (2.0, 7.0)	.064
SOFA score ≥ 5, n (%)	444 (55.1)	188 (71.8)	130 (53.5)	126 (41.9)	<.001
Medication or procedures, n (%)					
Vasopressor	216 (26.8)	101 (38.5)	73 (3.0)	42 (14.0)	<.001
IMV	365 (45.3)	169 (64.5)	108 (44.4)	88 (29.2)	<.001
Antiviral drugs	119 (14.8)	28 (1.7)	45 (18.5)	46 (15.3)	.044
Corticosteroids	331 (41.1)	110 (42.0)	92 (37.9)	129 (43.0)	.453
Heparin and its derivatives	646 (8.2)	215 (82.1)	199 (81.9)	232 (77.3)	.277
Outcome					
28-d mortality, n (%)	223 (27.7)	94 (35.9)	66 (27.2)	63 (2.9)	<.001

AIDS = acquired immune deficiency syndrome, BMI = body mass index, CCI = Charlson Comorbidity Index, COPD = chronic obstructive pulmonary disease, IMV = invasive mechanical ventilation, PaO_2_ = partial pressure of oxygen in arterial blood, RDW = red cell distribution width, SOFA = Sequential Organ Failure Assessment.

### 3.2. Serum calcium and mortality

To assess serum calcium levels and their association with 28-day mortality in COVID-19 patients, we applied 3 models: Model I: no adjustment; model II: adjustment for sex, race, age, and BMI; model III: further adjustment for RDW, partial pressure of oxygen in arterial blood, hypertension, diabetes, sepsis, CCI, antiviral drugs, vasoactive agents, and IMV. In the unadjusted model, a 1-unit increase in serum calcium corresponded to a 29% reduction in mortality (HR = 0.71, 95% CI = 0.61–0.82; *P* < .001, model I). Additional details can be found in Table 2. In model III, after accounting for potential confounders, serum calcium levels continued to show a significant association with 28-day mortality (HR = 0.83, 95% CI = 0.71–0.97, *P* = .021). Categorization into tertiles based on serum calcium levels revealed that the highest tertile (T3) had a significantly lower 28-day mortality risk than the lowest tertile (T1) (model III HR = 0.70, 95% CI = 0.50–0.99, *P* = .043). For the trend analysis, *P*-values were less than.05 across all 3 models.

**Table 2:** The relationship between serum calcium and 28-day mortality.

Variable	Total, N	Event, n (%)	Model I		Model II		Model III	
			HR (95% CI)	*P*-value	HR (95% CI)	*P*-value	HR (95% CI)	*P*-value
Calcium (mg/dL)	806	223 (27.7)	0.71 (0.61–0.82)	<.001	0.70 (0.61–0.80)	<.001	0.83 (0.71–0.97)	.021
Tertiles (mg/dL)								
T1 (1.5–7.9)	262	94 (35.9)	1.00 (Ref)		1.00 (Ref)		1.00 (Ref)	
T2 (8.0–8.4)	243	66 (27.2)	0.71 (0.52–0.97)	.030	0.70 (0.51–0.96)	.029	0.88 (0.64–1.22)	.451
T3 (8.5–11.3)	301	63 (2.9)	0.53 (0.38–0.72)	<.001	0.47 (0.34–0.65)	<.001	0.70 (0.50–0.99)	.043
*P*_trend_				<.001		<.001		.044

Model I: Unadjusted.

Model II: Adjusted for age, gender, race, body mass index.

Model III: Model II plus red cell distribution width, pressure of oxygen in arterial blood, hypertension, diabetes, sepsis, liver disease, chronic obstructive pulmonary disease, renal disease, asthma, acquired immune deficiency syndrome, Charlson Comorbidity Index, Sequential Organ Failure Assessment, antiviral drugs, corticosteroids, heparin and its derivatives, vasopressor, invasive mechanical ventilation.

CI = confidence interval, HR = hazard ratio, Ref = reference, T = tertiles.

### 3.3. Kaplan–Meier curves

**Figure 2.**
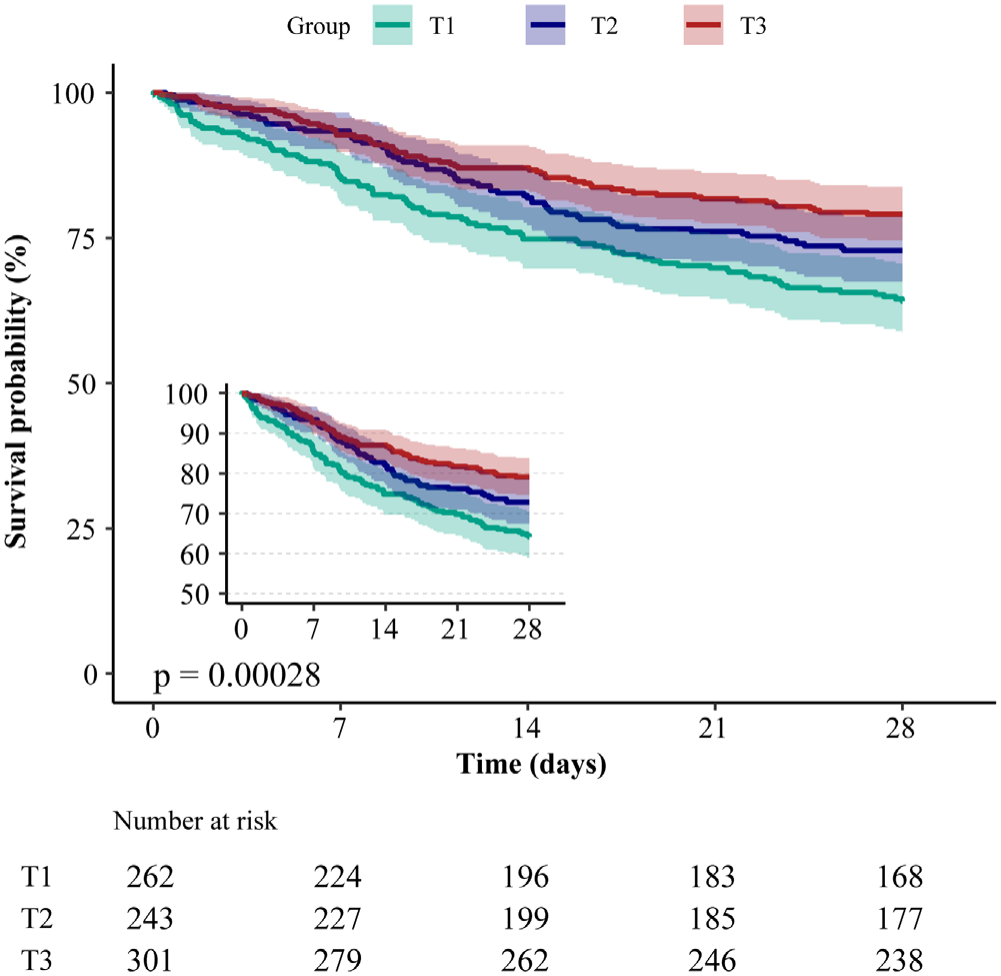
Kaplan–Meier survival curves for critically ill patients with COVID-19 based on serum calcium tertile. COVID-19 = coronavirus disease 2019, T = tertile.

Kaplan–Meier analysis showed a markedly higher 28-day survival rate for the T3 group versus the T1 and T2 groups, underscoring the significant variation in survival across the calcium level tertiles (*P* = .00028) (Fig. 2).

### 3.4. Subgroup analyses

**Figure 3.**
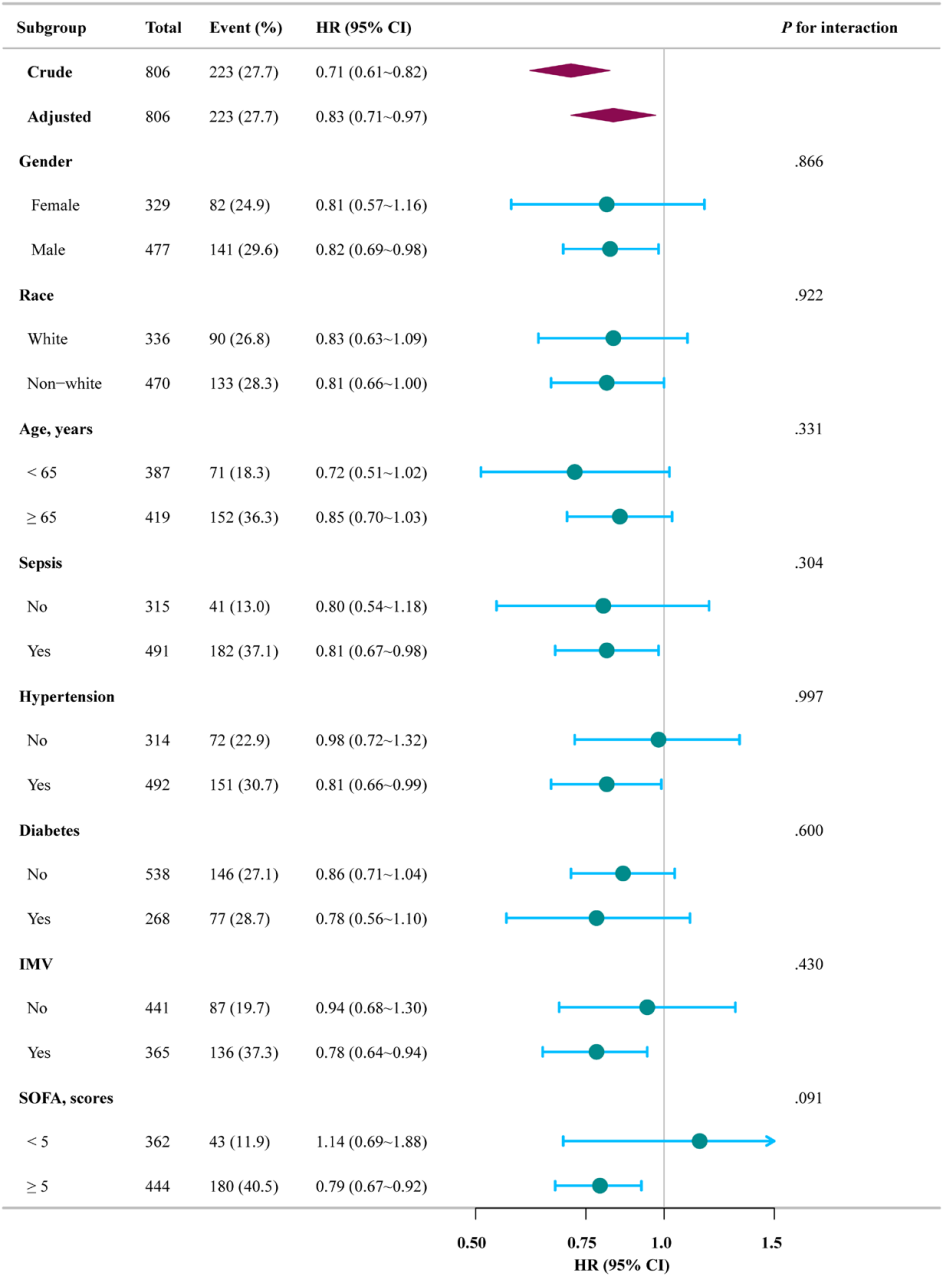
Subgroup analyses for the association of serum calcium with 28-day mortality in the critically ill patients with COVID-19. Adjusted for age, gender, race, body mass index, red cell distribution width, partial pressure of oxygen in arterial blood, hypertension, diabetes, sepsis, liver disease, chronic obstructive pulmonary disease, renal disease, asthma acquired immune deficiency syndrome, Charlson Comorbidity Index, SOFAscore (≥5 vs < 5), antiviral drugs, corticosteroids, heparin and its derivatives, vasopressor, IMV. CI = confidence interval, COVID-19 = coronavirus disease 2019, HR = hazard ratio, IMV = invasive mechanical ventilation, SOFA = Sequential Organ Failure Assessment.

A subgroup analysis was conducted to evaluate the consistency of the association between serum calcium levels and mortality, with participants categorized by age < 65 vs ≥ 65, gender, race, hypertension, diabetes, and IMV, and SOFA < 5 vs ≥ 5. Figure 3 illustrates the results in a forest plot. The analysis showed a uniform impact of serum calcium on mortality in all subgroups. No significant interactions between subgroups were found (*P* > .05).

## 4. Discussion

This retrospective cohort study found an inverse association between elevated serum calcium and mortality risk among severely ill COVID-19 patients requiring ICU transfer. After accounting for potential confounders, multivariate Cox regression analysis shows that higher serum calcium is associated with a lower risk of 28-day mortality in patients with severe COVID-19. The highest serum calcium group had a 30% lower risk of all-cause mortality compared to the lowest group. This study offers early insights into the connection between serum calcium and severe COVID-19 cases.

Limited studies have investigated how serum calcium levels relate to mortality in COVID-19 patients. Fatima et al^[23]^ demonstrated that lower serum ionic calcium concentrations are associated with higher mortality rates among COVID-19 patients. Bennouar et al^[24]^ conducted a study with 120 patients with severe COVID-19, and found that hypocalcemia was significantly tied to a higher risk of in-hospital death. A meta-analysis found a strong link between low serum calcium levels and higher mortality risk in COVID-19 patients (odds ratio = 6.99, 95% CI = 2.71–17.99).^[12]^ In our current research, our results showed that lower calcium levels were connected to increased fatality rates. Our conclusions are in line with the earlier findings, reinforcing the significance of early monitoring of serum calcium in individuals with COVID-19.

The precise physiological mechanism that underlies the link between serum calcium levels and clinical outcomes in COVID-19 cases remains unclear. Variations in serum calcium levels may stem from altered intestinal absorption, imbalances in the regulation by parathyroid hormone and vitamin D, or direct impacts caused by SARS-CoV-2 infection. Some studies indicate that cytokines may interfere with the production of calcium receptors, causing an imbalance in serum calcium levels. Severe illness is associated with significantly increased levels of proinflammatory cytokines, notably interleukin-1 and interleukin-6. The association between hypocalcemia and increased mortality rates might be attributed to the interplay.^[25,26]^ Severe illness is associated with significantly increased levels of proinflammatory cytokines, notably interleukin-1 and interleukin-6. The association between hypocalcemia and increased mortality rates might be attributed to the interplay between serum calcium levels and immune system function.^[27,28]^

This study possesses notable strengths. It leverages a large and substantial dataset from the MIMIC-IV database, a well-regarded resource for its high-quality real-world clinical data. In addition, the conduct of subgroup and sensitivity analyses strengthens the credibility of the results. However, our study is not without limitations. The retrospective design could potentially lead to selection bias. Furthermore, this study focuses on a single-center ICU population in the United States, which may not be generalizable to ICU patients with COVID-19 in other countries. This geographical and demographic specificity could limit the applicability of our findings to a broader, international context. In addition, calcium levels were recorded only at the time of ICU admission, preventing us from assessing the full trajectory of serum calcium changes over the course of the disease, which might affect the precision of our results. Last, the observational nature of the study precludes establishing a definitive causal relationship between serum calcium levels and COVID-19 outcomes. Future studies should use randomized controlled trials to establish causality and explore mechanisms linking calcium levels to COVID-19 outcomes, including immune function, inflammation, and cardiovascular health.

## 5. Conclusion

The study suggests that elevated serum calcium at admission is linked to a lower risk of 28-day mortality in individuals with critical COVID-19 infection. Further studies are needed to validate these findings and explore the underlying mechanisms.

## Acknowledgments

The authors thank Dr Qilin Yang of Department of Critical Care, The Second Affiliated Hospital of Guangzhou Medical University and the Physician Scientist Team for guidance on data extraction and analysis.

## Author contributions

**Formal analysis:** Jianmin Qu, Yingxiu Huang.

**Investigation:** Jianmin Qu, Yingxiu Huang, Lihua Shen.

**Methodology:** Jianmin Qu, Yingxiu Huang, Lihua Shen.

**Software:** Jianmin Qu, Yingxiu Huang.

**Supervision:** Jianmin Qu, Lihua Shen.

**Validation:** Jianmin Qu, Lihua Shen.

**Writing – original draft:** Jianmin Qu, Yingxiu Huang.

**Data curation:** Yingxiu Huang.

**Visualization:** Lihua Shen.

**Writing – review & editing:** Lihua Shen.
